# A Treatise on Spinal Irritation; the Result of Observations Made Principally in the General Hospital of Vienna

**Published:** 1845-01

**Authors:** 


### Art. X.

*Abhandlung über Spinal Irritation, &c.* Von Ludwig Türck, Doctor der Medicin, &c.—*Wien*, 1843.

*A Treatise on Spinal Irritation; the Result of Observations made principally in the General Hospital of Vienna.* By Dr. L. Türck, &c. —*Vienna*, 1843. 8vo, pp. 84.

What is spinal irritation? and what are its symptoms, course, nature, terminations, and treatment? Dr. Türck defines it as follows: "It has been observed in modern times that in certain nervous, and particularly hysterical conditions, a more or less extended portion of the spinal column was painful on pressure, and when touched with a hot sponge; and that these conditions were frequently cured, or at least relieved, by the abstraction of blood from, and the application of irritants to the painful vertebræ. Hence the opinion, that the nervous symptoms alluded to depended on an irritable condition of the spinal cord,—a condition expressed by the term *spinal irritation.*" It was by an extract from the publication of the Messrs. Griffin (Observations on the Functional Affections of the Spinal Cord, and Ganglionic System of Nerves, 1834), which appeared in Schmidt's Jahrbuch for 1838, that Dr. Türck's attention was drawn to the subject. He then instituted a series of researches, and often found occasion to correct, as he asserts, the vague statements of the English writers just mentioned.

Dr. Türck discriminates between symptomatic and idiopathic spinal irritation: the one being simply an accompaniment of other diseases; the other arising spontaneously, or persisting after the original affection has been removed. He names the increase of the pain, spasm, and other symptoms on pressing on the spinal column, reflexion; and the point, on which the pressure is applied, and these results follow, the reflex point. He adopts these terms because he thinks the reflex-theory will explain the pathological phenomena.

1. *Symptomatic spinal irritation.* In a case of caries of the molars and incisors, with inflammation of the jaws, the pain was reexcited by pressure on the second and third cervical vertebræ, and on the soft parts of the same sides as that affected. In a case of gastric fever, frontal and supra-orbital pain was excited through the four or five upper cervical vertebræ; in one case there was also a peculiar unpleasant sensation in the stomach, with an inclination to vomit; in the other, the unpleasant sensation of pressure in the stomach was excited through the last cervical vertebra, and first dorsal. In typhus fever, pressure on the upper half of the cervical vertebræ excited vertigo, tinnitus aurium, and headache. The sensation of dryness of the tongue and of thirst was excited in two cases by pressure about from the fourth to the sixth cervical vertebræ, and once the disagreeable taste experienced with coated tongue in the earlier stages, was entirely removed by this means. Twice in affections of the liver, the pain in the right shoulder and right clavicular region, and numbness of an extremity, were excited by pressure on the fourth and fifth dorsal vertebræ. These are the majority of the cases in which spinal irritation was a symptom of disease of the digestive organs.

The diseases of the heart and lungs present similar sympathetic phenomena. Irritation at the bifurcation of the bronchi was excited in catarrh and tubercular phthisis by pressure on from the third to the seventh cervical vertebra. In cases of pneumonia, it was not unfrequently observed that pain in the forehead and temples, weight in the frontal region, fulness of the head, and tinnitus aurium, were excited by pressure on the upper cervical vertebræ as far as the fifth. In one case, besides shooting and pain in the forehead, Dr. Türck observed impatience of light, and sensation of heat, and pressure on the eyes, to accompany pressure on the last cervical vertebræ. In many cases of pneumonia, difficulty of breathing and cough or pains under the sternum and ribs were excited by pressure on one or other of the dorsal vertebræ, or even by only gently pinching up the skin. In two instances, the tenderness on pressure extended over the whole abdomen, and in others pain along the false ribs, and numbness of the extremities were excited through pressure on the spinal column. These abdominal reflex phenomena were never observed in tubercular phthisis. The sensation of heat and tickling experienced in cases of hæmoptöe, could be re-excited by spinal pressure in two instances observed by Dr. Türck. In the one, the seat of this sensation (which immediately preceded the hæmoptöe) was on the left side of the lower half of the sternum, and it was re-excited by pressure on the upper dorsal vertebræ; in the other, it was seated in the region of the cartilages of the false ribs on the left side, and was brought on by pressure on the lower dorsal vertebræ. In pleuritis, as in pneumonia, the cephalic symptoms were reproduced by spinal pressure. Numbness of the extremity corresponding to the side affected could also be induced. In endocarditis and diseases of the valves of the heart, dyspnea could be brought on through the lower cervical and upper dorsal vertebræ.

Diseases of the blood also exhibit the phenomena of spinal reflexion. In a case of poisoning by digitalis, spectral flashes and muscæ volitantes could be excited through the last cervical vertebræ; in another, the appearance of green and yellow shoots before the eyes could be brought on by pressure on the cervical vertebræ from the second to the fifth. In diseases of the nervous system, this spinal irritation would of course be expected to appear as a symptom; various instances are related by Dr. Türck. He noticed it, too, in variola, in oophoritis, and in medullary sarcoma of the uterus.

Idiopathic spinal irritation was observed in intermittent fever during its three stages, in hysteria, neuralgia, cramp, and in the rheumaticforms of disease.

*Explanation of the phenomena of spinal irritation.* Spinal irritation may originate through the agency upon the spinal cord of incident excitor nerves coming from organs in a morbid state, or according to Stilling, in consequence of an abnormal condition of the capillary circulation of the spinal axis. This change in the spinal cord, in whatever way produced, is diffused through its substance, and thus engages the points of origin of other nerves, and so excites nervous symptoms, the *apparent* seat of which is the organs to which the nerves whose roots are so affected are distributed. It is in virtue of such a change as this at the roots of the dorsal sensitive nerves, that the state of spinal sensibility is induced. Dr. Türck sets forth reasons already stated by Stilling in support of the doctrine, that it is through the nerves of the surface and not by pressure on the spinal cord that the reflex phenomena are produced, and the pain experienced. We need not go over these reasons as they are exactly those stated by a recent English author. The practitioner must be ignorant indeed who, when he presses on the spinal column, can suppose for one moment that he is pressing, or directly irritating the spinal cord.

The seat of spinal irritation corresponds generally to the reflex point on the back. This is to be accounted for by the short course which the sensitive nerves of the surface of the spine have to run. There are cases, however, Dr. Türck mantains, in which the true seat of the irritation is not in the cord, but in the ganglia on the posterior roots, namely, those in which the excitor nerves are branches of the sympathetic. This he brings forward as something absolutely new, manifestly being ignorant (most happily for himself) that this doctrine has been known to English practitioners for many years.

Leaving the general pathology of s pinal irritation, Dr. Türck proceeds to trace the phenomena, according to the anatomical distribution and relations of the nerves. Thus, the shooting and pain in the forehead and temples, excited in cases of pneumonia by pressure on the three upper cervical vertebræ, are situate in the branches of the trigeminus, and Dr. Türck attempts to show from some experiments instituted by Magendie that the origin of this nerve is so low down as that portion of the medulla spinalis corresponding to the second cervical vertebræ. By similar arguments, other excited reflex phenomena are explained. Ocular spectra and amblyopia, together with pain in the head and eyes, are excited by pressure on the nape, and accounted for on the hypothesis that the roots of the trigeminus are irritated at their origin. The cephalic symptoms in dyspepsia are explained by an irradiation of irritation from the roots of the vagus to the roots of the trigeminus. It is not necessary to recount here the anatomical distribution of the vagus, and show how irradiation of irritation on its roots may give rise to dyspnea, cough, vomiting, cardialgia, &c. Nor need we pass, step by step, along the spinal and sympathetic nerves, and trace all the nervous symptoms originating in centric changes at the roots of the nerves. The story has been so often and so well told by English writers, that we can only wonder at the innocent ignorance of their works exhibited by the two most recent German authors on the subject, namely, Stilling and Dr. Türck. We must say, however, that they are much more excusable than those recent writers of our own nation, who bring forward crude essays on the subject, containing not one new fact or argument, and only remarkable as showing that their authors are entirely unacquainted with the physiology of the spinal cord and the pathology of spinal irritation.

We know not whether we rightly infer that because late English writers have displayed so much ignorance, English readers are ignorant, too, of the true pathology of a very numerous class of neuroses. We can state, however, with justice, that the doctrine of reflex function, developed by Dr. Hall, and by some of the best physiologists of our own time, has not yet entered so deeply into the minds of the profession at large, as to extensively modify their views of the pathology and treatment of a certain class of cerebro-spinal affections, or to influence their practice; and this we presume to be a sufficient reason for placing the subject in *extenso* before our readers.

The incident action of the nerves on the spinal cord and brain, whether in exciting or paralysing, has long been known. Whytt inferred, from some experiments with opium on frogs and dogs, that "not only those nerves, to which opium is immediately applied, are rendered incapable of performing their office, but that the brain, spinal marrow, and whole nervous system are affected in the same manner, *solely* by the action of the *opium* on the nerves which it touches. For its effects upon dogs are too instantaneous to allow of the supposition, that the more subtile parts of this poison are received into the blood, and by that means are conveyed to the brain; and in frogs, after the heart is taken out, and consequently a stop put to the circulation, yet a solution of opium, injected into the stomach and intestines, has the same effect, as when these animals are entire." We quote from his 'Remarks on the Sympathies of the Nerves,' and he details many "sympathies" of the nerves, as, for example, "the contraction of the pupil is not owing to light acting as a stimulus on the iris, but solely to the sympathy between this membrane and the retina," or in Hallian phraseology, the retina is incident excitor to the motor nerve of the iris. And it is to be observed, that although he does not explain, nor attempt to explain, the mode in which this sympathy or *connexion in function* occurs, he makes no error in the classification of his facts. The principle of irradiation of sensation will explain them all.

In stating his views, Whytt was by no means original. Hoffmann, Boerhaave, and others, his predecessors and contemporaries, had previously explained and illustrated them. Whytt's merit consisted in making the theory lucid and the facts consistent. We need not refer to the physiological views of Haller, Unzer, or Prochaska. The quotation which serves as a motto to the publication of the Messrs. Griffin will show how distinctly the doctrine of reflex or centric pathological action was expressed by an early writer of this century. "I wish explicitly to state," says Burns, in his 'Principles of Midwifery,' "that many diseases, supposed to arise from local causes acting directly on the organs affected, do often proceed from disordered states, or preternatural excitement of some portion of the spinal cord. Even inflammation of these organs may be thus produced." The "preternatural spinal excitement" of Burns is simply the spinal irritation of more modern pathologists. A succession of writers holding similar views might be made out. We would only mention Abernethy, Brown, Copland, Billing, Tate, Teale, Marshal, Darwall, William and Daniel Griffin, and Laycock, as the principal English authors of this class. Compared with the doctrines of these writers, we pronounce Dr. Türck's explanation hypothetical, meagre, and unsatisfactory, and in doing this we hold ourselves bound to produce a better. We would first, however, revert to certain established principles in neurology. Pain may be produced in various ways. First, the termination of the sensitive nerves may be so altered by mechanical or physical changes, that pain shall be excited. Thus act the capillary tension in inflammatory affections, and the disruption of the molecules of the nerve by pricking, tearing, burning, &c. But the change thus begun at the terminating molecule must pass on along the trunk of the nerve from molecule to molecule, until it arrives at the central axis; the whole chain is brought into operation. That is one and the most common mode in which pain is produced. But the painful change may be commenced in any part of the course of the nerve; either on the fibril or within the ganglia through which it passes: in this case the painful sensation is referred, not to the point at which the change commences, but to the point at which the nerve terminates. It is thus that persons who have had a limb amputated, and subsequently a morbid growth on the cut end of the nerve, have experienced the most excruciating neuralgia and painful convulsion, apparently in, and referred to, the skin and muscles of the limb long since severed from the trunk.

The morbid change in the course of the fibril may not be, however, so extensive as to cause pain, *per se*; it most usually is not; but it is just so great as to magnify ordinary impressions and render them painful; it acts as a physiological magnifier. It is thus that in cases of spinal irritation, the slightest touch—not pressure,—but the touch of a feather will excite exquisite pain, and this followed by reflex acts. That the cutaneous nerves of the back are often in this highly sensitive condition from some physiological change in their course, and not at the periphery, is undoubted; but we strongly object that the dorsal sensitive nerves are not exclusively, or even generally, in this morbidly sensitive state. The subcutaneous tubercle, a small knob on a nerve, is an example of this kind, for the slightest touch will often induce general convulsions. Not unfrequently in cases of hysteria, there is a tender spot on the middle of the sternum, and pressure here induces a convulsive gasp: or there is a tender spot on the vertex, or some circumscribed spot of the scalp, pressure on which excites various nervous symptoms. The mere sensual nerves, as the optic, olfactory, and auditory, are also often in this "affectible" state, so that colours, as red and black, sweet odours, and acute sounds will induce general convulsions, and other reflex phenomena. In this case, the doctrine, of the true spinal system are inapplicable, because, according to this, the brain and cerebral nerves are inexcitor; it is most manifest, however, that such cases cannot be explained by the doctrine of exclusively *spinal* irritation.

If we carefully analyse the symptoms observed in well-marked cases of spinal irritation, we shall find that they can all be elucidated on the supposition, that there is a centric change so operating as to exalt the functions of the cerebro-spinal axis. This change can scarcely be considered strictly structural, because we know that structural changes are marked by anæsthesia, or paralysis: on the other hand, we know that the circulation may be the medium through which the actual change is induced. Opium renders frogs tetanic, as strychnine acts on man and lower animals; the slightest touch of the surface in poisoning by strychnine is sufficient to excite convulsions. The poison of the rabid dog so exalts the functions of the incident excitor and reflex motor nerves of the respiratory and spinal ganglia, that a breath of air on the face will excite a convulsive gasp and general spasms. In "spinal irritation" the change is probably in the capillary circulation: 1, of the cerebro-spinal axis; 2, of the ganglia of the sympathetic, or ganglia of the posterior spinal nerves; and 3, of the fibrils of the nerves themselves. This purely functional derangement of the nervous system has been termed neuræmia by Dr. Laycock, one of the recent English writers on this subject before mentioned, and the class of diseases to which it gives origin, neuræmic. (British and Foreign Medical Review, vol. XI, p. 66.)

It will thus be seen that the exciting causes of the phenomena of cerebro-spinal irritation may originate diversely. The exaltation of function of the cerebro-sp nal ganglia may be induced, firstly, by the reaction of diseased viscera upon the central axis. It is thus that the cramps in diarrhea, and other intestinal affections, and paroxyms of insanity or delirium are induced. Secondly, the membranes of the spinal cord may be congested, or in such a morbid state as to act on that portion of the axis, as meningeal excitement acts on the brain. It is extremely probable that this is a frequent cause of spinal irritation in persons of an arthritictaint. Thirdly, the nerves passing from the spinal axis, or the ganglia or the posterior roots, may be liable to an influence similar to that just mentioned; and in either case, with results not unlike those of irritation of the cord proper. Fourthly, a morbid condition of the blood circulating through the central axis, may be the exciting cause of the chain of morbid phenomena. This morbid state may itself depend on various causes. It may depend on deficient depuration from a deficient action of excretory organs, as the kidneys, liver, or colon; or on the entrance of a poisonous matter into the circulation from without, or the blood may be in an abnormal state, in consequence of defective assimilation or, as frequently happens, from sudden and profuse sanguineous discharges.

Extensive and complicated as the etiology of neuræmic diseases may appear, a thorough acquaintance with their varied forms, and with the anatomy and physiology of the nervous system renders their diagnosis, prognosis, and treatment comparatively easy. In no class of affections, however, is the experienced eye more necessary than in this; their physiognomy must be known and studied. Symptoms implicating the motor or sensitive nerves are seen in all cases of disease; and the first step in diagnosis is to determine whether they be of central or peripheral origin. There may be exquisite sensibility to light, or great tenderness of the abdomen, for example, and these symptoms may depend on either a peripheral affection of the nerves of the conjunctiva, or of the peritoneal surface, or on a centric affection of the brain, or of the nerves of the abdominal surface. The one is inflammatory, the other neuræmic, and the treatment utterly dissimilar. The diagnosis is all-important: in an example of this kind, an error either way may be fatal.

If we were to attempt an illustration of our remarks as to the diagnosis, we could not take a more apt instance than that of abdominal tenderness. Where it depends on spinal irritation, it will be found that the history of the patient presents instances of her having previously suffered from neuræmic affections. The affectible state of the cutaneous nerves of the abdomen is never observed to occur alone, the nerves of the abdominal viscera suffer also. The kidneys, for example, secrete less or more urine than natural: if less, the deficiency amounts occasionally to complete ischuria; if more, the urine is pale and diabetic. And so there is one or other of the two opposite states of constipation and diarrhea, but more usually constipation, with spasm of the colon, giving rise to colic. In the more aggravated cases, the motor nerves of the large intestines, bladder, abdominal parietes, and lower extremities, are also affected; and tympanites, vesical paralysis, constipation, and paraplegia ensue. The tenderness experienced is not simply tenderness on pressure, but it is a tenderness to the slightest touch, and when there is spinal tenderness, for it is not always present in these cases, the tenderness is of the same kind. We believe there is much error current on this point: writers continually writing about "tenderness on pressure," in cases of (the so-called) spinal irritation, when in fact the tenderness is manifest on the touch of a feather. The abdominal tenderness of peritoneal or visceral inflammation differs altogether from the preceding, both in its history, and concomitant symptoms. It is rarely seen in neuræmic females, except when the cause is quite manifest, as for example, when there is chronic structural disease of the peritoneum or abdominal viscera, accompanied by inflammation, or when it appears in parturient females as a symptom of metritis. We believe the neuræmic state is rarely coincident with structural disease within the abdomen, or terminates in it.

The accompanying phenomena differ widely: in the tenderness from peritoneal inflammation it is impossible not to be struck by the peculiarly haggard expression of countenance of the patient; in neuræmic tenderness, the expression is rather that of peevishness and pain than of mortal suffering. In the latter, there is rarely or never, the grass-green vomit observed in peritonitis; the pulse too is fuller, rounder, and larger, nor are the knees of the patient drawn up as in peritoneal inflammation: in the latter, tenderness on pressure, and not tenderness to the touch, is the distinguishing characteristic.

The diagnosis of all neuræmic affections, whether their seat lies in the head, thorax, or abdomen, is, *mutatis mutandis*, precisely similar. The previous history of the patient, and the physiological character of the symptoms themselves will almost always enable the practitioner to decide. Inflammatory diseases of the heart, lungs, and brain, are peculiarly liable to be confounded with neuræmic affections of those organs. The violent palpitations and coughs, the latter singularly varied as to "timbre," according to the portion of the pulmonary mucous membrane affected, and the deficient respiration, the cephalœa, syncope, &c., accompanying them are often most alarming to the practitioner, and seem to call urgently for the most active treatment. And yet probably a counter-irritant, or an endermic stimulant or sedative, with a slight antispasmodic combined with an aperient or diuretic, and perfect quietude, will suffice to dissipate all these alarming symptoms in a few hours. The rule we should recommend in cases of this kind is to depend chiefly on the *causal* diagnosis; by diligent inquiry to ascertain whether the individual has been exposed to any sufficient cause of inflammation, and if not, and if also the marks or causes of a generally neuræmic state be detected, to temporize and watch. In persons already debilitated by excessive loss of blood or profuse discharges, or who have been ailing with unaccountable complaints for a length of time, abstraction of blood, except the most moderate, we believe injurious even in affections truly inflammatory, and tends to increase the congestion we wish to remove. When the brain is the seat of neuræmia, local or general bleeding is absolutely wrong. With respect to the treatment of cases of cerebro-spinal irritation we would above all observe, that it must be constitutional. They principally occur, idiopathically, in females whose parents are gouty, and of the nervous temperament, or at least who have themselves suffered from affections of the nervous system. Neuræmia of the cerebral and spinal ganglia is not unfrequently seen in females, one or other of whose parents has been insane. This we believe is extremely usual, and the fact is of importance both in the diagnosis and treatment of the affections of all the viscera in connexion with the spinal cord. While remedies suitable to the constitutional peculiarities of the case are administered, endermic medication, on the principle of incident excitor action, will be found of singular benefit; even the metallic neurines, as arsenic, may be thus employed with safety and efficiency. We would conclude this short sketch of the treatment, by the observation that no plan whatever will be successful unless the hygienic condition of the patient, namely, the condition as to mental occupation, sleep, diet, pure air, sufficient light, and exercise be strictly attended to. Remedies should be administered as subsidiary to the attainment of those essential conditions to recovery. Moral treatment in all its branches is peculiarly adapted to cases of this kind, as the brain rarely escapes irritation, and in some instances insane cunning, infirmities of temper, and even nymphomania and other forms of insanity are developed.

---

